# Awareness and Prevalence of Mycotoxin Contamination in Selected Nigerian Fermented Foods

**DOI:** 10.3390/toxins9110363

**Published:** 2017-11-08

**Authors:** Ifeoluwa Adekoya, Patrick Njobeh, Adewale Obadina, Cynthia Chilaka, Sheila Okoth, Marthe De Boevre, Sarah De Saeger

**Affiliations:** 1 Department of Biotechnology and Food Technology, University of Johannesburg, Doornfontein 2028, South Africa; obadinaw@gmail.com; 2 Department of Food Science and Technology, Federal University of Agriculture, Abeokuta 2240, Nigeria; 3 Laboratory of Food Analysis, Department of Bioanalysis, Ghent University, Ghent B-9000, Belgium; adaku80@yahoo.com (C.C.); marthe.deboevre@ugent.be (M.D.B.); sarah.desaeger@ugent.be (S.D.S.); 4 Department of Botany, School of Biological Sciences, University of Nairobi, Nairobi 00100, Kenya; dorisokoth@yahoo.com

**Keywords:** fermented foods, mycotoxins, awareness, food safety, LC-MS/MS

## Abstract

Fermented food samples (*n* = 191) including maize gruel (*ogi*), sorghum gruel (*ogi-baba)*, melon seed (*ogiri*), locust bean (*iru*) and African oil bean seed (*ugba*) from Southwest Nigeria were quantified for 23 mycotoxins, including aflatoxin B_1_ (AFB_1_), fumonisin B_1_ (FB_1_), and sterigmatocystin (STE) using liquid chromatography-tandem mass spectrometry. The practices, perceived understanding and health risks related to fungal and mycotoxin contamination amongst fermented food sellers was also established. Data obtained revealed that 82% of the samples had mycotoxins occurring singly or in combination. FB_1_ was present in 83% of *ogi-baba* samples, whereas 20% of *ugba* samples contained AFB_1_ (range: 3 to 36 µg/kg) and STE was present in 29% of the *ogi* samples. In terms of multi-mycotoxin contamination, FB_1_ + FB_2_ + FB_3_ + STE + AFB_1_ + alternariol + HT-2 co-occurred within one sample. The awareness study revealed that 98% of respondents were unaware of mycotoxin contamination, and their education level slightly correlated with their level of awareness (*p* < 0.01, *r* = 0.308). The extent to which the analyzed mycotoxins contaminated these food commodities, coupled with the poor perception of the population under study on fungi and mycotoxins, justifies the need to enact fungal and mycotoxin mitigation strategies along the food chain.

## 1. Introduction

Processing of food relies on a series of preservative technologies developed to enhance quality, safety, and acceptability, one of which is fermentation. Fermentation is the oxidation of carbohydrates to produce a wide range of products principally alcohol, organic acids and carbon dioxide through microbial activities [1]. Fermentation being a low-cost technology improves the digestibility and functionality of foods and facilitates food detoxification [2]. So far, most microorganisms involved in the fermentation of foods (cereals, legumes, oil seeds, etc.) belong mainly to the *Lactobacillus, Leuconostoc*, *Lactococcus*, *Pediococcus*, *Bacillus* and *Saccharomyces* genera. *Iru* is a condiment that is produced via the fermentation of African locust bean (*Parkia biglobosa*) by *B. substilis*, *B. licheniformis* and *B. pumilis* [3], whereas *ogiri* is from melon (*Citrullus colocynthis*) seed with *Bacillus*, *Escherichia* and *Pediococcus* spp. as the fermenting organisms [3]. The solid-state alkaline fermented proteinous product of the African oil bean seed (*Pentaclethra macrophylla*) is known as *ugba* [4], while *ogi* is a product of lactic acid fermentation of maize or sorghum and principally consumed as weaning food. *Ogiri* and *ugba* like *iru*, are principal condiments used to flavor stews and soups [5]. *Ugba* is also consumed as snack and used in the preparation of porridge.

These products amongst variants such as *injera*, *banku*, *amasi*, *fufu*, *garri*, *kenkey*, *uji*, and *mawe* are indigenous to Africa. In Africa, they are typically manufactured in homes under spontaneous conditions with little or no process control [2]. Their production is also dominated by informal processing sectors (cottage and rural small-scale processors) that make use of different traditional processing methods thereby, bringing about variation in substrates used, processing conditions (time, temperature, moisture etc.), packaging materials, handling and storage practices [6]. These factors determine the quality and safety of the final products. However, irrespective of the processing method employed, it is expedient for food marked for sale to be of good quality and free from pathogenic and spoilage microorganisms such as fungi and associated toxins.

For fungi, a number of strains belonging mainly to the *Aspergillus*, *Fusarium* and *Penicillium* genera that attack various food commodities are toxigenic, producing various types of mycotoxins. About 25% of the global food output is contaminated by mycotoxins, causing significant economic losses [7]. Moreover, they are a serious health hazard, as they are known to be carcinogenic, nephrotoxic and immunotoxic. Mycotoxins of significance in sub-Saharan Africa (SSA) in terms of health and economy are fumonisins (FBs), aflatoxins (AFs), ochratoxin A (OTA), zearalenone (ZEN), and trichothecenes (TCs) [8]. They have been found present in different food categories mainly in cereals such as maize and oil seeds such as melon that are substrates used in the production of fermented foods. Therefore, the presence of mycotoxins in fermented foods (i.e., *iru*, *ogi*, *ogi baba*, *ugba* and *ogiri*) cannot be undermined. Even though fermentation could play a role in the degradation or detoxification of mycotoxins in foods, there are increasing reports of mycotoxins in fermented foods [9,10,11]. In Nigeria, there are no in-depth studies that report multiple mycotoxin contamination in the selected fermented foods, hence the need for this study.

On the other hand, a model proposed for the management of mycotoxins in SSA, identified awareness creation and enlightenment of people on mycotoxins as a principal strategy that can contribute to limit mycotoxin contamination in foods [12]. However, in recent years, increasing reports on the prevalence of multiple mycotoxins in foods consumed in SSA [8,9,11,13,14,15,16,17] suggests that only minimal efforts are deployed towards mycotoxin management particularly amongst food processors and sellers. Though, their main goal is to generate income, chances are high that adequate understanding of health implications of mycotoxin contamination through experiential learning will prompt behavioral changes and the enactment of necessary mitigation actions. Strategies such as sorting of moldy grains, utilization of adequate packaging materials, proper drying, implementation of appropriate storage methods and facilities can reduce mycotoxin contamination to a large extent. It is therefore crucial to investigate the practices, understanding and perceived health risks of fungal and mycotoxin contamination amongst stakeholders along the food chain including fermented food sellers to ascertain the level of awareness. It was also imperative to establish the magnitude to which multiple mycotoxins contaminate the fermented foods offered for sale. In a clear context, the objective of this study was to determine the level of awareness of fungal and mycotoxin contamination amongst selected fermented food sellers in Southwest Nigeria and to assess the level of mycotoxin contamination in the products (*iru*, *ogiri*, *ogi*, *ogi-baba* and *ugba*) they offer for sale.

## 2. Results and Discussion

### 2.1. Perception Studies

An appraisal was carried out on fermented food sellers perceived attitudes, practices and knowledge of fungal colonization of foodstuffs, being an antecedent of mycotoxins in Southwest Nigeria. The result demonstrated a wide knowledge gap amongst those under study (*n* = 86), as 98% could not link fungi to mycotoxin contamination and perceived associated health risks. However, these findings corroborate those of other studies [14,18,19]. According to Siegrist and Cvetkovich [20], a significant number of people in both developed and developing nations are not well informed on contaminants in foods. Majority (93%) of participants were females (*n* = 80) as shown in Table 1, which accentuates the role of women under the spotlight of food production and processing in Africa. Amongst the respondents, 57% store their finished products in polyethylene bags and 20% in leaves for an average of seven days. Fermented foods stored in leaves are more predisposed to fungal and mycotoxin contamination because of the indigenous microflora of the leaves and the deployment of little or no effort to clean or sterilize the leaves before use. In the study of Adegunloye et al. [21], *Thaumatococcus daniellii* and *Musa paradisiaca* leaves which are popularly used in packaging or storing fermented foods had high fungal load and toxigenic fungi such as *A. niger*, *A.flavus* and *P. expansum* were prevalent. Although, fermented foods are perceived to be safe, their mode of storage could predispose them to fungal and mycotoxin contamination if the storage methods are not complemented by other means of preservation.

The sellers (95%) obtained foods from different sources (markets or processors) as retailers while a few process their products themselves, the products were also stored over varying length of time e.g., up to seven days for finished products and up to three months for raw materials. Storage and marketing practices employed amongst the sellers also have the tendency to facilitate variations in mycotoxin contamination [22]. Knowledge of environmental factors such as humidity, temperature, insect infestation, pre- and post-harvest practices that affect fungal growth and mycotoxin production in foods are particularly important when developing and implementing strategies for the control of fungi and mycotoxins along the food chain [23]. It was evident in the study that the majority of study participants (92%) could attribute these factors to the persistence of fungi in foods.

Amongst the respondents, 22% expressed that they frequently experience fungal contamination. Fungi can thrive on varieties of foods but some foods are better substrate for their growth than the others. For example, *ugba* will favor the growth of fungi based on its alkaline pH than *ogi* which has an acidic pH [24]. Therefore the contrast amongst the respondents in terms of the frequency of contamination is expected. *Ugba* and *ogiri* were observed to be more susceptible to fungal contamination amongst the foods but were still offered for sale. *Ogiri* upon fungal invasion had characteristic black color while *ugba* overgrown with mold were posited to be more suitable as an ingredient for porridge than its principal use as condiment. Particularly worthy of note was the willingness expressed by 97% of the respondents to attend training on mycotoxin mitigation. Public awareness trainings drives attitudinal transformation if target groups have confidence in the lessons received and apprehend the problem well enough to be persuaded to revise old practices and habits [25].

Few respondents had no formal education (11%), whereas most of those under study had primary education (61%). Table 2 presents important information on the association between the level of education and knowledge on fungi and mycotoxins amongst respondents. Their knowledge of fungi correlated positively (*p* < 0.01, *r* = 0.355) with their ability to identify foodstuffs contaminated with fungi which could be due to their experience of fungal contamination as shown in Table 2. Moreover, findings also revealed that the level of education had a significant but slightly positive influence (*p* < 0.01, *r* = 0.296) on their apprehension of fungi and mycotoxin contamination (*p* < 0.01, *r* = 0.308). Dosman et al. [26] highlighted that individuals with higher education levels are likely to more knowledgeable and aware of some food contaminants than individuals with less education because they have more access and tend to seek for more information on food safety and related issues [27]. Also in Nigeria, studies have posited that educational attainment is crucial for public awareness of food safety [28]. For individuals with little or no formal education as observed in this study, more strength lies with this as an easily transmittable skill since mycotoxin related issues are not precisely covered in the curricular of any primary or secondary school in Nigeria as the case may be for other countries in Africa and beyond. Even though our findings reveal that education level correlates positively with awareness, knowledge and recognized benefits, it is expedient to make the problem known to all categories of individuals.

Mycotoxins are at the forefront amongst chronic food toxicants [19], usually occurring below levels that elicit acute health effects, but such levels could provoke long-term health implications amongst humans and animals [29]. It may therefore be difficult to associate several health complications to mycotoxin exposure, which strongly supports the poor perception on the subject demonstrated by respondents in this study. In addition to this, because mycotoxin can be present in foods after the dissipation of fungi, it was therefore unexpected for respondents to physically discern a food that is contaminated with mycotoxins in addition to fact they seem not to alter the taste or flavor of foods. These variations need to be considered and communicated particularly during the implementation of trainings aimed at fungal and mycotoxin mitigation.

The findings of our research have profound implications for strategies directed towards mycotoxin management in fermented foods and other food categories in Nigeria and SSA, based on studies [12,25,28] that have established that awareness and education are critical elements in reducing the menace of mycotoxins in developing countries. The study was particularly targeted towards fermented food sellers but has extensive inference for the curtailment of food hazards/contaminants in evolving economies. It was therefore imperative to further establish the extent of mycotoxin contamination in fermented foods (i.e., *ogi*, *ugba*, *iru*, *ogiri*, and *ogi-baba*) based on the feasibility of human exposure revealed through the poor perception of mycotoxin contamination amongst their sellers.

### 2.2. Method Performance Characteristics

Table 3 shows the results of the method validation parameters including the LOD, LOQ and AR of the different fermented food matrices. The calibration curves for the analytes were linear and the AR of all the analyzed mycotoxins varied between 89% and 109%, which aligns within the range set by the EC [30]. The LODs for 3-ADON 15-ADON, AFB_1_, AFB_2_, AFG_1_, AFG_2_, DAS, and ROQ C were <6 μg/kg while the LOQs of STE, and ZEN were ≤20 μg/kg in all the tested fermented food.

### 2.3. Mycotoxin Contamination

In this study, the multi-mycotoxin profile of Nigerian fermented food products including *ogiri*, *ugba* and *iru*, intended for use as condiments, as well as *ogi* and *ogi-baba*, popularly consumed as breakfast cereals, was delineated (Table 4). Generally, 56% of the 34 fermented food samples positive for AFB_1_ had levels above the maximum regulatory limit of 2 µg/kg in foods according to the EC [31]. Data obtained via LC-MS/MS showed that *ogiri* samples had a higher incidence of AFB_1_ (48%) (range: 3–4 µg/kg) compared to others. Co-occurrence of AFB_2_ and AFG_2_ was established in *ogiri*. We also noted the prevalence of these important analogues AFs (including others, i.e., AFB_1_ and AFG_1_), singly or in combination in all the samples, which might be due to the presence of AF-producing fungi in similar samples of *ogiri*, *ugba*, *ogi*, *ogi-baba* and *iru* [24]. It has been established that chronic exposure to AF from fermented foods affects close to 4.5 billion persons in the developing countries [32]. The presence of *Aspergillus* spp. and AF in some raw materials used in the manufacture of the tested fermented foods has been previously established. Ezekiel et al. [33] recovered AFB_1_ and total AF from melon seeds used in the manufacture of *ogiri* at a mean level of 37.5 µg/kg and 142 µg/kg, respectively, meanwhile Makun et al. [15] reported the presence of *A. flavus* and AFB_1_ in 54% of sorghum for *ogi baba* production. The biosynthetic pathway of AFB_1_ has also been studied [9] with averantin, averufin, norsolorinic acid, versicolorin A and STE established as precursors/intermediate compounds. STE and AFB_1_ are synthesized by the same *Aspergillus* spp. and the presence of AF in the fermented foods can as well be attributed to the presence of such a precursor as STE. STE was detected in *ugba* (range: 22–27 µg/kg) and *ogi* (range: 4–7 µg/kg), was <LOQ in *ogiri* and *ogi* and absent in *iru*.

The vulnerability of food products such as maize to OTA contamination worldwide is documented [16]. The data presented in this study show OTA being present in *ogi-baba*, *iru*, and *ugba*, at mean levels of 6, 6, and 9 µg/kg, respectively. OTA was not found in *ogi*, which is contrary to the report of Oyelami et al. [17] on the presence of OTA in maize-based foods. All the *ugba* and *ogiri* samples were positive for OTA, containing levels that were above the 5 µg/kg recommended for foodstuffs by EC [31]. OTA is a potent secondary metabolite synthesized in foods by more than ten fungal species with *A. ochraceus* and *P. verrucosum* as principal producers in the tropics and in the temperate regions, respectively [34]. The same toxin is associated with kidney and liver impairment, Balkan endemic nephropathy, oxidative DNA damage and has been classified by the International Agency for Research on Cancer (IARC) [35] as a Group 2B carcinogen. ROQ C is often regarded as one of the most important fungal contaminants in fermented foods and beverages [36] and in this study, it was only detected in *iru* and at a low incidence rate (range: 10–14 µg/kg). Being a potent neurotoxin at high concentrations above 1500 µg/kg [31], it can therefore be deduced that the same samples are considered safe for consumers in terms of ROQ C concentrations. Like AF, STE, and OTA, the occurrence of ROQ C in these analyzed foods could be due to the participation of various fungi during fermentation, which is principally by chanced inoculation. Odunfa and Adeyele [37] identified *Aspergillus* and *Penicillium* fungi during the fermentation of *ogi-baba*.

The TCs are a large family of over 150 chemically related toxins produced principally by the *Fusarium* genera [38]. Based on their core structures, they are classified into four types: A, B, C and D. Type A includes mainly T-2 toxin and HT-2 toxin together with NEO, and DAS in this list. DON, NIV, 3-ADON, 15-ADON and FUS-X are the Type B TCs mycotoxins. In relation to toxicity according to Schollenberger et al. [39], type A TCs are more toxic when compared to type B TCs. In terms of geographical locations Type A TCs are not commonly reported in Africa, but in our study, the type A TH-HT-2 was the most frequently occurring, and it levels in *ogi* (range: 20–21 µg/kg) exceeded the recommended level of HT-2 + T-2 (15 µg/kg) in infant foods by EC [40]. Concerning *iru*, 9 samples were positive for HT-2 within the range of 17 to 51 μg/kg (mean: 33 μg/kg) (Table 4) and T-2 was also detected in *iru.* Generally, T-2 and HT-2 toxins are of great concern based on their capacity to induce oxidative stress, inhibit DNA, RNA, and protein synthesis as well as mitochondrial performance [40]. Furthermore, the contamination of both TCs mycotoxins can co-occur together with DAS, because of similarity in biosynthesis at the side branch of the pathway of T-2 [41], which was only observed in *ogiri.* The level of DON in all the positive samples (*n* = 18) reach a maximum of 118 µg/kg, which was far less than the maximum limit for DON in processed cereals (750 µg/kg). The frequency and contamination level found for the acetylated derivative of DON (3-ADON) was low, whereas no analyzed samples contained 15-ADON. Chilaka et al. [11] found much higher contamination levels of DON and 3-ADON than any reported in our study.

The FBs were the dominant mycotoxins in *ogi* and *ogi-baba*, with most of the *ogi* samples having FB_1_ and FB_2_ higher than maximum set limit for FB_1_ + FB_2_ (200 µg/kg) in maize-based infant foods by EC [31]. This suggests the high exposure of infants to FB_1_ and FB_2_. The high incidence of this toxin group observed in *ogi*, a maize-based product substantiates the vulnerability of the maize crops to FB producing fungi such as *F. proliferatum* and *F. verticillioides.* Additionally, the low levels of FBs observed in *ogi baba* as a fermented processed sorghum product also correlates well with the findings that sorghum is less susceptible to fungal infestation when compared to maize [11]. Whilst, data on the occurrence of the studied mycotoxins in *ogi baba* is scarce, studies from Nigeria have reported the presence of FBs in sorghum grains [11,15]. Amongst the alkaline fermented food studied, only *iru* (7%) was positive for FB_1_, FB_2_ and FB_3_ with mean values of 113, 38 and 84 µg/kg, respectively. FB_1_ has been linked to liver and esophageal cancer, and on that note was classified as a group 2B carcinogen [35]. Recently in Tanzania, Shirima et al. [42] studied child growth during early childhood in relation to AF and FBs exposure and established that exposure to FBs alone or together with AF is a factor of growth impairment in children. As established in our study, *ogi* as a weaning food in Nigeria can be considered unsafe for consumption by infants.

**Figure 1:**
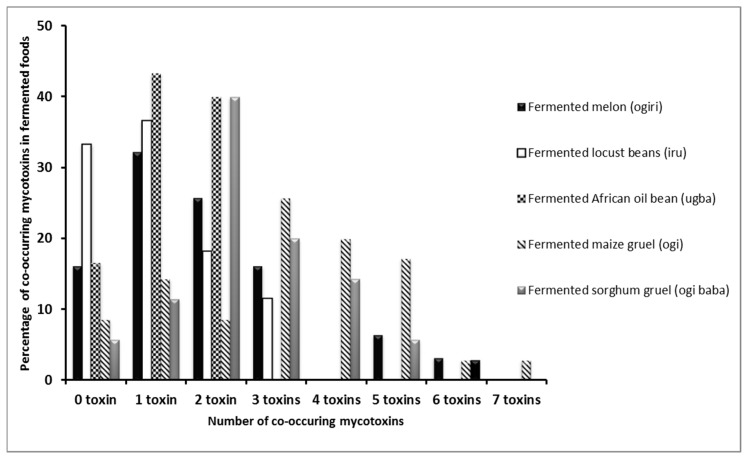
Percentage co-occurrence of mycotoxins in fermented foods from Southwest Nigeria.

In 90% of the samples positive for ZEN (*n* = 20), levels recovered were less than 50 µg/kg, which is insignificant when compared with the maximum limit of 50–1000 µg/kg in foods in 16 countries where ZEN is being regulated [43]. According to Kpodo et al. [9], ZEN is largely produced by some *Fusarium* spp. in cool dry climates between 10 °C and 15 °C, whereas temperatures from 27 °C to 40 °C commonly persist yearly in Nigeria. Howbeit, the low levels of ZEN detected in the samples may be due to the persistence of such climatic conditions that are unfavorable for the production of the toxin in Nigeria. Amongst all these fermented food types, 18% (*n* = 35) was devoid of the tested mycotoxins, *ugba* was the least contaminated in terms of the number of mycotoxins (10) found compared to others including *ogiri* (11), *ogi-baba* (12), *ogi* (13) and *iru* (16). *Ugba* is encapsulated in extremely hard seed coats, which make it less prone to fungi and mycotoxin contamination than others with less formidable coats. This might account for the low levels in the number of mycotoxins found in *ugba* in comparison with other samples. Co-occurrence of several mycotoxins within the fermented foods analyzed was also observed (Figure 1).

Out of the 191 samples analyzed, 82% (*n* = 156) had mycotoxins occurring singly or in combination. For the 23 different mycotoxins analyzed in each food matrix, 3 different toxins co-occurred in 16% *ogiri*, 12% *iru*, 26% *ogi* and 26% *ogi* samples tested. This phenomenon has been demonstrated for several mycotoxins in foods consumed in Africa [11,14,15]. DON usually co-occurs with its acetylated forms but this was not the case in this study. The co-occurrence of DON and ZEN has also been established, but with ZEN mostly occurring at a lower concentration than DON [38]. This relationship was observed in some *ogiri* samples that were positive for both mycotoxins. Also, the co-occurrence of up to seven metabolites (FB1 + FB_2_ + FB_3_ + STE + AFB_1_ + AOH + HT-2) in *ogi* could be due to its susceptibility to fungal contamination when compared to other samples. The multiple mycotoxins observed within the same sample could exacerbate the health risks amongst humans since they can elicit some synergistic and additive effects especially at levels above those accepted by various regulatory bodies.

The varying contamination levels, incidence and co-occurrence of mycotoxins in the fermented foods analyzed are a result of many cogent factors. It however, remains unclear whether the mycotoxins recovered from the samples analyzed are due to carry-over from the raw materials used in processing them, or due to fungal contamination of processed foods tested in this study. In any case, ignorance as well as climatic conditions that prevail in the study sites, seems to also play a role. Unacceptable trade activities of mixing moldy food products with high-quality products to maximize profit also persists due to non-enforcement of regulatory limits on locally grown crops or locally produced products sold in the markets, and ignorance on the existence of mycotoxins. The importance of the right processing environment and conditions cannot be underrated as most of these foods were manufactured in homes under unhygienic conditions. Besides an unhygienic processing environment, improper storage practices, which provide optimal conditions for mould development and subsequent mycotoxin accumulation, may exacerbate the situation. Bearing in mind that, these fermented products are not the only dietary sources of mycotoxin exposure amongst humans, the overall daily exposure to these mycotoxins can be high.

## 3. Conclusions

This study gave an insight into the safety of fermented foods produced in Nigeria and equally established the awareness of the sellers towards fungal and mycotoxin contamination and associated health risks. We observed that there exists a wide knowledge gap amongst participants on this aspect of food safety. In terms of the food analyzed, a significant fraction of the samples (156/191) had mycotoxins occurring singly or in combination though relatively at low incidence and contamination levels. *Ogi* was the most contaminated sample based on the total number of samples positive (94%, *n* = 35) for the analyzed toxins which makes the risk of mycotoxin exposure higher amongst its consumers. Some of the samples exceeded the maximum limit for FB, AF, OTA and ZEN in foods as regulated by the EC. In broad terms, the incidence of type A TCs was slightly higher than type B TCs. All the samples were negative for 15-ADON. AOH, AME, STE, ENN B and ROQ C were also present at low levels in few samples. *Ogi-baba* and *ogi* had the highest number of co-occurring fungal metabolites. To the best of our knowledge, this is the first study that assessed the presence of mycotoxins in *ugba* and *ogi-baba*. As well as the first to report a wide range of previously unreported mycotoxins in *iru, ogiri* and *ogi* consumed in Nigeria.

Considering the high level of consumption of these fermented foods in Nigeria, strategies towards mycotoxin mitigation should be a priority. Awareness needs to capture good agricultural practices aimed at reducing fungal infestation of the raw materials during growth and storage, while executing training on ways of selecting high-quality raw materials. Hands-on learning activities needs to be integrated with awareness campaigns to create more opportunities for the target groups to adopt the recommendations provided. Awareness can also be created from a gender focal point where women being at the forefront of food production are involved in the formulation of education programs on mycotoxin management. Proper understandings of the economic and health effects of mycotoxins are important drivers as individuals are most likely to take steps towards mycotoxin reduction if effects are known. Proposed strategies should therefore emphasize benefits. To pave the way forward, there is need for enforcement of risk-based food laws, encouragement of dietary diversity, sustained use of intervention technologies and more surveillance programs that could be implemented to provide toxicological and exposure data.

## 4. Materials and Methods

### 4.1. Sampling

The cluster sampling method was used to obtain fermented foods namely; maize gruel (*ogi*), sorghum gruel (*ogi-baba*), locust bean (*iru*), African oil bean seed (*ugba*) and melon seed (*ogiri*) from various fermented food sellers in Southwest Nigeria between February 2015 and July 2016. Composite samples of each fermented food: *ogi* (*n* = 35), *ogi-baba* (*n* = 35), *iru* (*n* = 60), *ugba* (*n* = 30) and *ogiri* (*n* = 31) were taken to obtain a total of 191 composite samples. Each composite sample of about 270 g was an aggregate of sub samples obtained from three different fermented food sellers. All samples were collected in sterile containers and immediately transported to the laboratory. Afterwards, each composite sample was properly mixed and trisected twice to obtain a representative sample of 30 g, after which they were stored at −18 °C for mycotoxin analysis at the Laboratory of Food Analysis, Ghent University, Belgium.

### 4.2. Awareness Studies

A descriptive cross-sectional study was carried out amongst some of the fermented food sellers within the sampling area in February 2015 using a questionnaire that consisted of closed- and open-ended questions. The questionnaire (Table S1) was designed to capture the demographics, practices, understanding and perceived health risk of food contamination by fungi and mycotoxins amongst fermented food sellers. The sellers were informed about the purpose of the study and their verbal consent was obtained with a standardized consent form. In total, 86 respondents willingly participated in the study.

### 4.3. Mycotoxin Analysis

#### 4.3.1. Materials and Chemicals

Methanol, glacial acetic acid, and acetonitrile (LC-MS/MS grade) were purchased from Biosolve B.V. (Valkenswaard, The Netherlands). Ammonium acetate and acetic acid (analytical grade) were supplied by Merck (Darmstadt, Germany). HPLC grade methanol and n-hexane in addition to Whatman^®^ (Maidstone, UK) glass microfiber filters were obtained from VWR International (Zaventem, Belgium). Ultrafree-MC centrifugal filter devices (0.22 μm) were obtained from Millipore (Brussels, Belgium). MultiSep^®^226 AflaZon+ immunoaffinity columns and C18 solid phase extraction (SPE) columns were obtained from Romer Labs (Gernsheim, Germany) and Grace Discovery Sciences (Lokeren, Belgium), respectively. Water was purified in a Milli-Q Gradient apparatus (Millipore, Brussels, Belgium). All other reagents and chemicals were of analytical grade.

#### 4.3.2. Mycotoxin Standards

Mycotoxin standards comprising of aflatoxin B_1_ (AFB_1_), aflatoxin B_2_ (AFB_2_), aflatoxin G_1_ (AFG_1_), aflatoxin G_2_ (AFG_2_), fumonisin B_1_ (FB_1_), fumonisin B_2_ (FB_2_), deepoxy-deoxynivalenol (DOM), 15-acetyl-deoxynivanelol (15-ADON), neosolaniol (NEO), OTA, alternariol (AOH), alternariol monomethyl ether (AME), zearalenone (ZEN), nivalenol (NIV), deoxynivanelol (DON), 3-acetyl-deoxynivanelol (3-ADON), sterigmatocystin (STE), roquefortine C (ROQ C), enniatin B (ENN B), fusarenon-X (FUS-X), HT-2 toxin (HT-2) and zearalanone (ZAN) were obtained from Sigma-Aldrich (Bornem, Belgium). Fumonisin B_3_ (FB_3_) was procured at Promec Unit (Tynberg, South Africa), while T-2 toxin (T-2) and diacetoxyscirpenol (DAS) were purchased from Biopure Referenzsubstanze (Tulln, Austria).

#### 4.3.3. Sample Preparation

All samples were dried in a hot air oven (UM200, Memmert, Schwabach, Germany) and milled to a particle size between 0.5 and 1 mm. Milled samples were accurately measured (5 ± 0.005 g), and reinforced with internal standards (ZAN-10 µg/mL and DOM-50 µg/mL), and allowed to equilibrate for 15 min in the dark. Extraction of mycotoxins in the sample was done using 20 mL of acetonitrile/acetic acid/water (79/1/20, *v*/*v*/*v*). The mixture was vortexed for 10 s, placed on an overhead shaker (Agitelec, Paris, France) for 1 h and centrifuged for 15 min at 3500 rpm. All the supernatant was transferred into a pre-conditioned SPE C18 column and defatted (2×) using 10 mL n-hexane. Two cleanup procedures were applied to recover the 23 mycotoxins. First, 27.5 mL of acetonitrile/acetic acid (99/1, *v*/*v*) was added to 12.5 mL of the defatted extract, and passed through a MultiSep 226 AflaZon+ immunoaffinity column. Second, using a glass micro filter (General Electric, Coventry, UK), 2 mL of defatted extract was filtered and combined with the MultiSep 226 eluate and evaporated to dryness. The residue was reconstituted in 150 µL of injection solvent consisting of methanol/water/acetic acid (57.2/41.8/1, *v*/*v*/*v*) and 5 mM ammonium acetate (0.385 g/L). The reconstituted extract was placed in an Ultrafree^®^ PVDF centrifuge filter (Merck, Darmstadt, Germany), and centrifuged at 10,000 rpm for 10 min. The eluent was transferred into an LC-MS/MS injection vial prior to analysis.

#### 4.3.4. Liquid Chromatography-Tandem Mass Spectrometry

A Waters Acquity UPLC apparatus paired to a Quattro Premier XE Tandem Mass Spectrometer (Waters, Milford, MA, USA) was utilized for the identification and quantification of the analytes. Data acquisition and processing utilities included the use of the MassLynx™ (V. 4.1) and QuanLynx^®^ (V. 4.1) software (Micromass, Manchester, UK). The column used to separate the analytes of interest was a Symmetry C18 column (150 mm × 2.1 mm i.d. 5 μm particle size) with a guard column (10 mm × 2.1 mm i.d.) of the same material (Waters, Zellik, Belgium). The chromatographic conditions set were similar to those of Ediage et al. [44]. Mobile phase A contained acetic acid/methanol/water (1/5/94, *v*/*v*/*v*) and 5 mM ammonium acetate (0.385 g/L), and mobile phase B contained acetic acid/water/methanol (1/2/97, *v*/*v*/*v*) and 5 mM ammonium acetate (0.385 g/L). With a sample injection volume set at 10 μL, the total analytical run time was 28 min with a pressure that varied between 0 and 5000 psi. The mass spectrometer was operated using selected reaction monitoring (SRM) channels in positive electrospray ionization (ESI+) mode. Further details on the mycotoxin transitions are reported by De Boevre et al. [45] and Monbaliu et al. [46]. For the identification of the targeted mycotoxins, the criteria of the Commission Regulation 657/2002/EC [47] were followed.

#### 4.3.5. Method Validation

The Commission Regulation 401/2006/EC [30] was used for the validation studies. Variables including limit of quantification (LOQ), limit of detection (LOD) and apparent recovery (AR) were accessed by spiking mycotoxin-free samples (blank) with the different mycotoxins in triplicates. ZAN and DOM were used as internal standards and matrix matched calibration curves (MMC) were constructed from the ratio of the peak area of each analyte to the internal standard. The linearity of each analyte was estimated graphically using a scatter plot, and the linear regression model evaluated using a lack-of-fit test, while apparent recoveries were established by dividing the calculated concentration by the theoretical concentration.

### 4.4. Data Analysis

A descriptive statistics (mean, range, frequencies, and percentages) of the data generated in this study was performed using Microsoft Office Excel 2010 (Redmond, WA, USA). In addition, the degree of awareness of fungal and mycotoxin contamination amongst the fermented food sellers was correlated with their level of education using Kendall’s tau-b test on SPSS version 23.0 (IBM Corporation, NY, USA).

## Figures and Tables

**Table 1:** Descriptive statistics and knowledge of fungal and mycotoxin contamination amongst fermented food sellers.

Parameters	Incidence (%)	Parameters	Incidence (%)	Parameters	Incidence (%)
**Sociodemographic Variables**					
***Gender***		***Education level***		***Age***	
Male	6 (7)	None	9 (11)	<30 years	6 (7)
Female	80 (93)	Primary	52 (61)	31–50 years	74 (86)
		Secondary	23 (27)	>50 years	6 (7)
		Tertiary	2 (2)		
**Fermented Food Characteristics**					
***Mode of consumption***		***Food type***		***Food source***	
Direct consumption	28 (33)	*Ogi*	28 (33)	Home processed	4 (5)
Food Ingredient	46 (54)	*Iru*	21 (24)	Market	32 (37)
Both	12 (14)	*Ogiri*	19 (22)	Processors	50 (58)
		*Ugba*	18 (21)		
**Storage Variables**					
***Storage method of raw materials***		***Storage duration of raw materials***		***Average shelf life of raw material***	
Bags	13 (15)	1–3 months	13 (15)	1–4 weeks	1 (1)
Containers	5 (6)	>3 months	5 (6)	>4 weeks	17 (20)
Not applicable	68 (79)	Not applicable	68 (79)	Not applicable	68 (79)
***Storage method of finished product***		***Storage duration of finished product***		***Average shelf life of finished product***	
Polyethylene bags	49 (57)	1–7 days	86 (100)	1–3 days	14 (16)
Containers	14 (16)	>7 days	-	3–7 days	42 (49)
Paper	3 (4)			>7 days	30 (35)
Leaves	17 (20)				
Wooden Boxes	3 (4)				
**Knowledge of Fungi and Mycotoxins**					
***Knowledge of fungi***		***Identification of fungal contamination in food***		***Frequency of contamination***	
Yes	63 (73)			Rarely	36 (42)
No	16 (19)	Yes	59 (68)	Frequently	19 (22)
Not sure	7 (8)	No	27(32)	Not applicable	31 (36)
***Perception of reasons of fungi occurrence***		***Knowledge of health risks associated with fungal contamination***		***Knowledge of production of toxins by fungi***	
Storage	21 (24)	Yes	7 (8)	Yes	3 (4)
Bad raw materials	19 (22)	No	79 (92)	No	83 (96)
Insect infestation	18 (21)				
All of the Above	21 (24)				
Not sure	7 (8)				
***Knowledge of mycotoxin contamination***		***Willingness to attend training on mycotoxin mitigation***			
Yes	2 (2)	Yes	83 (97)		
No	84 (98)	No	3 (3)		

Number of respondents: 86.

**Table 2:** Kendall’s tau-b correlation between education and awareness level of fungi and mycotoxins amongst respondents.

Correlations	Level of Education	Do You Know What Fungi Is	Can You Identify Food with Fungi	Does Fungi Contamination of Foodstuffs Cause Health Problems	Do You Know Fungi Produce Toxins	Have You Heard of Mycotoxin Contamination
Level of Education	1.000	0.296 **	−0.172	0.014	0.048	0.308 **
Do you know what fungi is	0.296 **	1.000	0.355 **	0.249 *	0.075	−0.139
Can you identify food with fungi	−0.172	0.355 **	1.000	0.069	0.190	0.100
Does fungi contamination of foodstuffs cause health problems	0.014	0.249 *	0.069	1.000	0.122	0.221 *
Do you know fungi produce toxins	0.048	0.075	0.190	0.122	1.000	0.109
Have you heard of mycotoxin contamination	0.308 **	−0.139	0.100	0.221 *	0.109	1.000

** Correlation is significant at *p* < 0.01 (2-tailed); * correlation is significant at *p* < 0.05 level (2-tailed); *n*: number of respondents (86); values along each column are correlation coefficients.

**Table 3:** Method performance parameters of the fermented food matrices.

Mycotoxins	Calibration Range µg/kg	Fermented Melon (*ogiri*)			Fermented Locust Bean (*iru*)			Fermented African Oil Bean (*ugba*)			Fermented Maize Gruel (*ogi*)			Fermented Sorghum Gruel (*ogi baba*)		
		LOD	LOQ	AR	LOD	LOQ	AR	LOD	LOQ	AR	LOD	LOQ	AR	LOD	LOQ	AR
Deoxynivalenol	200–800	11	22	100	4.9	9.8	99	15	30	101	7	14	97	12	24	101
Nivalenol	100–400	48	96	100	11	22	100	21	42	103	35	70	101	87	175	99
Neosolaniol	50–200	20	40	95	16	32	99	24	48	96	2.2	4.4	103	3.0	6.0	100
Fusarenon-X	100–400	39	78	97	8.1	16	101	25	50	96	21	42	100	45	90	100
3-Acetyldeoxynivalenol	25–100	2.3	4.6	96	2.0	4.0	102	1.2	2.4	101	5.0	10	105	12	24	97
15-Acetyldeoxynivalenol	12.5–50	1.7	3.5	94	3.9	7.9	101	1.8	3.7	96	10	20	95	7.0	14	99
Aflatoxin B_1_	10–40	2.0	4.0	96	1.2	3.3	96	1.5	3.0	100	3.8	7.5	100	5.0	10	100
Aflatoxin B_2_	10–40	2.3	4.6	96	1.8	3.3	94	1.4	2.8	96	1.8	3.5	99	2.5	5.0	102
Aflatoxin G_1_	10–40	3.9	7.8	99	1.7	3.3	95	1.9	3.9	98	1.8	3.5	98	2.5	5.0	101
Aflatoxin G_2_	10–40	3.7	7.4	96	1.2	2.3	91	2.2	4.4	94	3.8	7.5	100	5.0	10	99
Diacetoxyscirpenol	2.5–10	0.9	1.8	97	0.7	1.4	97	1.0	2.0	89	0.3	0.6	99	0.5	1.0	94
Alternariol	50–200	6.5	13	98	9.7	20	100	5.9	11	98	40	80	92	40	80	99
Alternariol Methyl Ether	100–400	54	107	96	5.0	10	96	4.6	9.2	98	5.0	10	109	6.3	12	96
HT-2 Toxin	50–200	6.5	13	98	7.4	14	98	15	30	94	6.5	13	85	6.5	13	95
T-2 Toxin	50–200	12	24	98	14	28	94	13	26	100	3.6	7.2	87	8.0	16	94
Fumonisin B_1_	200–800	24	48	97	22	44	100	38	76	97	8.2	16	87	10	20	98
Fumonisin B_2_	200–800	11	22	99	9.4	18	99	43	87	95	12	23	89	11	22	100
Fumonisin B_3_	25–100	13	26	97	21	42	97	33	66	94	14	28	89	14	28	96
Ochratoxin A	25–100	11	22	89	1.2	2.4	93	3.6	7.2	90	1.5	3.0	99	2.5	5.0	95
Sterigmatocystin	25–100	5.5	11	100	1.7	3.3	97	1.9	3.8	95	1.3	2.5	100	2.5	5.0	101
Roquefortine C	5–20	4.9	9.7	101	1.2	2.3	99	1.0	2.0	99	4.0	8.0	97	6.0	12	98
Zearalenone	50–200	9.8	20	96	2.9	5.9	92	4.4	8.8	104	3.3	6.5	102	3.8	7.6	93
Enniatin B	40–160	26	52	93	6.4	13	94	5.6	11	99	6.3	12	82	7.9	16	91

LOD: limit of detection (µg/kg); LOQ: limit of quantification (µg/kg); AR: Apparent recovery (%).

**Table 4:** Contamination of mycotoxins (µg/kg) in fermented foods from Southwest Nigeria.

Mycotoxins	Fermented Melon (*n* = 31) (*ogiri*)			Fermented Locust Bean (*n* = 60) (*iru*)			Fermented African Oil Bean (*n* = 30) (*ugba*)			Fermented Maize Gruel (*n* = 35) (*ogi*)			Fermented Sorghum Gruel (*n* = 35) (*ogi baba*)		
	% +ve	Range	Mean	% +ve	Range	Mean	% +ve	Range	Mean	% +ve	Range	Mean	% +ve	Range	Mean
Deoxynivalenol	3 (10)	<LOQ-54	31	4 (7)	<LOQ-118	62	4 (13)	36–38	37	4 (11)	<LOQ-55	32	3 (9)	32–112	60
Nivalenol	0	0	0	0	0	0	0	0	0	0	0	0	3 (9)	<LOQ	<LOQ
Neosolaniol	1 (3)	0	<LOQ	0	0	0	0	0	0	0	0	0	0	0	0
Fusarenon-X	0	0	0	3 (5)	40–76	62	0	0	0	0	0	0	0	0	0
3-Acetyldeoxynivalenol	0	0	0	1 (2)	0	19	0	0	0	0	0	0	0	0	0
15-Acetyldeoxynivalenol	0	0	0	0	0	0	0	0	0	0	0	0	0	0	0
Aflatoxin B_1_	15 (48)	3–4	<LOQ	1 (2)	0	6	6 (20)	3–36	20	8 (23)	<LOQ-17	<LOQ	4 (11)	10–24	11
Aflatoxin B_2_	5 (16)	<LOQ	<LOQ	1 (2)	0	<LOQ	1 (3)	0	<LOQ	7 (20)	<LOQ-7	6	0	0	0
Aflatoxin G_1_	0	0	0	2 (3)	8–8	8	0	0	0	2 (6)	0	0	1 (3)	0	16
Aflatoxin G_2_	2 (7)	<LOQ	<LOQ	3 (5)	3–6	4	0	0	0	0	0	0	0	0	0
Σ Aflatoxins	17 (55)	3-12	4	7 (12)	3–8	5	7 (23)	3–36	18	9 (26)	<LOQ-17	8	4 (11)	<LOQ-40	16
Diacetoxyscirpenol	1 (3)	0	1	0	0	0	0	0	0	0	0	0	4 (11)	1–2	1
Alternariol	0	0	0	0	0	0	0	0	0	3 (9)	<LOQ	<LOQ	0	0	0
Alternariol Methyl Ether	6 (19)	64–153	115	6 (10)	19–77	38	4 (13)	25–193	109	0	0	0	8 (23)	30–35	33
HT-2 Toxin	4 (13)	18–35	27	9 (15)	17–51	33	1 (3)	0	17	3 (9)	20-21	21	0	0	0
T-2 Toxin	0	0	0	7 (12)	28–31	29	1 (3)	0	15	0	0	0	0	0	0
Fumonisin B_1_	0	0	0	4 (7)	61–167	113	0	0	0	25 (71)	68–2492	384	14 (40)	<LOQ-68	39
Fumonisin B_2_	0	0	0	4 (7)	32–42	38	0	0	0	23 (66)	94–659	250	9 (25)	<LOQ-65	34
Fumonisin B_3_	0	0	0	4 (7)	76–89	84	0	0	0	18 (51)	42–404	112	26 (74)	<LOQ-42	28
Σ Fumonisin B_1_, B_2_	0	0	0	8 (13)	32–167	76	0	0	0	25 (71)	68–3151	645	18 (52)	<LOQ-129	48
Σ Fumonisin B_1_, B_2_, B_3_	0	0	0	12 (20)	32–167	78	0	0	0	27 (77)	42–3555	672	29 (83)	<LOQ-168	55
Ochratoxin A	6 (19)	<LOQ-27	<LOQ	7 (12)	<LOQ-21	9	1 (3)	0	9	0	0	0	2 (6)	5–6	6
Sterigmatocystin	6 (19)	<LOQ	<LOQ	0	0	0	2 (7)	22–27	25	10 (29)	4–7	4	8 (23)	<LOQ	<LOQ
Roquefortine C	0	0	0	2 (3)	10–14	12	0	0	0	0	0	0	0	0	0
Zearalenone	8 (25)	21–45	33	5 (8)	11–33	18	4 (13)	39–117	72	3 (9)	<LOQ	<LOQ	0	0	0
Enniatin B	0	0	0	0	0	0	4 (13)	<LOQ	<LOQ	5 (14)	12–14	13	0	0	0

Only concentrations higher than LOQ are recorded and samples containing concentrations higher than LOD were considered positive/contaminated; % +ve: percentage of positive samples.

## Supplementary Materials

The following are available online at www.mdpi.com/2072-6651/9/11/363/s1, Table S1: Questionnaire on Demographics, Practices, Understanding and Perceived Health Risk of Fungal and Mycotoxins Contamination amongst Fermented Food Sellers in Nigeria.
