# Supplementary material for: Awareness and Prevalence of Mycotoxin Contamination in Selected Nigerian Fermented Foods
Source: Toxins (Basel). 2017 Nov 8;9(11):363. doi: 10.3390/toxins9110363 (PMC5705978; doi:10.3390/toxins9110363)
Supplement: Supplementary file 1 [file toxins-09-00363-s001.pdf]

Questionnaire on Demographics, Practices, Understanding and Perceived Health Risk of Fungal and Mycotoxins Contamination amongst Fermented Food Sellers in Nigeria

(Instruction: Tick as appropriate)

Food Type:

Location:

1. Socio-demographic Variables

a) Gender
Male ☐ Female ☐

b) Age
<30 Years ☐ 31-50 Years ☐ >50 Years ☐

c) Education Level

None ☐ Primary ☐ Secondary ☐ Tertiary ☐

2. Fermented Food Characteristics

a) Fermented food source

Home processed ☐ Purchased in the market ☐ Purchased directly from processors ☐

b) Storage duration of raw materials of the fermented food

1-3 Months ☐ >3 Months ☐ Not Applicable ☐

c) Storage duration of fermented food before sale

1-7 Days ☐ >7 Days ☐

d) Storage method of raw materials of fermented food

Bags ☐ Containers ☐ Not Applicable ☐

e) Storage method of fermented food before sale

Polyethylene bags ☐ Containers ☐ Paper ☐ Leaves ☐

Wooden boxes ☐

f) Average shelf life of raw material of the fermented food

1 Month ☐ >1 Month ☐ Not Applicable ☐

g) Average shelf life of the fermented food

1-3 Days ☐ 3-7 Days ☐ >7 Days ☐

h) Mode of consumption

Directly ☐ As food ingredient ☐ Both ☐

3. Knowledge of Fungi, Mycotoxins and Health Effects

S/N	Questions	Yes		No
1	Do you know what fungi are?			
2	Can you identify food/crops contaminated with fungi?			
3	Do you experience fungal contamination in the fermented food you offer for sale?			
4	How frequent do you experience fungal contamination?	Rarely	Frequently	Not Applicable
5	Why do you think fungal contamination occur?			
6	Does fungal contamination of foodstuffs cause any health problem when contaminated foods are consumed?			
7	Do you know fungi produce toxins called 'mycotoxins'?			
8	Do you know the health effect associated with mycotoxin contamination?			
9	Are you willing to attend training on mycotoxins and how they can be reduced in our foods?			

4. Any other comment or suggestion(s)

THANK YOU
